# A Retrospective Evaluation of Serum Symmetric Dimethylarginine Concentration in Dogs With Protein‐Losing Enteropathy

**DOI:** 10.1111/jvim.70068

**Published:** 2025-03-24

**Authors:** Yeon Joon Park, Alexander J. German, David Brewer, Erin O'Connell

**Affiliations:** ^1^ Institute of Infection, Veterinary and Ecological Sciences University of Liverpool Neston UK; ^2^ Institute of Life Course and Medical Sciences University of Liverpool Neston UK

**Keywords:** chronic enteropathy, gastrointestinal disease, GFR, renal biomarker

## Abstract

**Background:**

Serum symmetric dimethylarginine (SDMA) is abnormally increased in people with inflammatory bowel disease (IBD). Changes in dogs with gastrointestinal disease, such as protein‐losing enteropathy (PLE), have not been assessed.

**Objectives:**

Evaluate SDMA concentration in non‐azotemic dogs with PLE.

**Animals:**

A total of 127 client‐owned dogs, 17 with PLE, 34 controls matched for age, breed, sex, and neuter status, and 76 additional controls for multiple linear regression modeling.

**Methods:**

Retrospective case–control study. The clinical records of a United Kingdom referral hospital were reviewed. Dogs with azotemia or prior glucocorticoid or immunosuppressive treatment were excluded. Dogs diagnosed with PLE that had serum symmetric dimethylarginine (SDMA) concentrations measured were compared with the matched controls. Signalment, clinical presentation, clinicopathological abnormalities, treatment, and SDMA concentration pre‐ (PLE‐T0) and post‐ (PLE‐T1) treatment were recorded.

**Results:**

At baseline, SDMA concentration was higher in PLE (T0, 15.2 ± 2.02 μg/dL) than in control (11.0 ± 3.13 μg/dL) dogs (*p* < 0.001; Hedge's G, 1.48), but decreased with treatment (PLE‐T1: 10.3 ± 2.78 μg/dL; T0 vs. T1: *p* = 0.01, Hedge's G, 1.31). Serum creatinine concentration was similar in PLE (T0, 0.81 ± 0.24 μg/dL) and control (0.85 ± 0.26 μg/dL) dogs at baseline (*p* = 0.57; Hedge's G, 0.18). Serum albumin concentration was lower in PLE (1.60 ± 0.51 g/dL) than in control (2.96 ± 0.49 g/dL) dogs (*p* < 0.001; Hedge's G, 2.68) before treatment, but increased with treatment (PLE‐T1: 2.29 ± 0.65 g/dL; T0 vs. T1: *p* = 0.003; Hedge's G, 1.14), although it remained lower than the concentration in controls (*p* = 0.002; Hedge's G, 1.23). No other clinicopathological differences were evident.

**Conclusions and Clinical Importance:**

Serum SDMA concentration is increased in dogs with PLE; the clinical relevance of this finding requires further investigation.

**Abbreviations**
BCS: body condition score
BIC: Bayesian information criterion
CBC: complete blood count
GFR: glomerular filtration rate
IBD: inflammatory bowel disease
MCS: muscle condition score
NO: nitrous oxide
PLE: protein‐losing enteropathy
PRMT5: protein arginine methyltransferase 5
SD: standard deviation
SDMA: symmetric dimethylarginine
UPCR: urine protein‐to‐creatinine ratio

## 1 Introduction

Protein‐losing enteropathy (PLE) is a syndrome of various gastrointestinal diseases characterized by excessive enteric protein loss and increased protein turnover, with substantial mortality and relapse rates [1, 2].

Serum symmetric dimethylarginine (SDMA) concentration has been a promising renal biomarker for dogs since its initial validation, including good correlation with glomerular filtration rate (GFR) in dogs, resulting in its introduction into small animal clinical practice [3, 4, 5, 6, 7]. Compared with serum creatinine concentration, changes in SDMA concentration might be more sensitive at detecting early loss of renal function [5, 6]. Furthermore, SDMA is not affected by muscle mass or cachexia, which often occurs in chronic kidney disease (CKD) and PLE [8, 9, 10]. However, as a by‐product of protein methylation in all nucleated cells [11], SDMA may be sensitive to increased protein turnover. To date, evaluation of extrarenal factors affecting SDMA in dogs has been limited. Multicentric lymphoma has been associated with increased SDMA concentration and increased protein turnover, probably secondary to altered protein metabolism [12].

In human medicine, SDMA concentration is increased in conditions associated with decreased GFR, such as CKD and congestive heart failure, but also in inflammatory conditions including glaucoma and polycystic ovary syndrome [13, 14, 15], where an extrarenal mechanism is suggested. Furthermore, inflammatory bowel disease patients have increased serum SDMA concentrations, again likely because of increased protein turnover [16, 17, 18]. From these findings, it has been suggested that SDMA may be a potential therapeutic target for people with inflammatory bowel disease.

Based on these observations, we hypothesized that dogs diagnosed with PLE, without concurrent azotemia, would have increased serum SDMA concentrations. Our primary objective was to determine if SDMA concentration is increased in affected dogs. Secondary objectives included identifying which animal and clinicopathological variables were associated with changes in SDMA concentration in dogs with PLE, and assessing if SDMA concentration changed with successful treatment.

## 2 Materials and Methods

### 2.1 Study Animals

Medical records from the University of Liverpool Small Animal Teaching Hospital from November 2017 to December 2020 were retrospectively evaluated for dogs diagnosed with PLE, henceforth referred to as “PLE cases.” The following eligibility criteria were used: history and clinical signs compatible with PLE (vomiting, diarrhea, weight loss, or altered appetite for > 3 weeks in duration), as previously described [19]; CBC, serum biochemistry, and SDMA concentrations performed during the study period, and evidence of hypoalbuminemia (albumin concentration < 2.63 g/dL, as previously defined [20]). Cases that had evidence of a hepatic or renal condition, based on results of CBC, serum biochemistry, abdominal imaging, or urinalysis (> 1+ protein on urine dipstick or urine protein creatinine ratio [UPCR] > 0.5) were excluded. Cases also were excluded if hypoadrenocorticism was suspected or if the histopathologic diagnosis was neoplastic in nature. Dogs were not eligible if serum SDMA concentration was measured > 2 weeks before or after initial presentation, if azotemia was present (serum creatinine concentration ≥ 1.4 mg/dL), or if glucocorticoid or immunosuppressive medication had been administered in the 3 months before presentation.

An a priori power analysis was conducted to determine the minimum sample size required for the study group using the pwr package from an online open‐access statistical environment used for all analyses (R, version 4.3.1 [21, 22]; see below). Using Pearson's correlation coefficient obtained from the comparison of SDMA in human IBD patients (*R*_p_ = 0.45) [17], an alpha of 0.05 and a power of 95%, a study sample size of 20 dogs and matched‐control sample of 40 dogs were required. The study protocol was reviewed and approved by the Veterinary Research Ethics Committee at the University of Liverpool (VREC1161a), and all owners provided written consent for the use of anonymized data from their dogs' clinical records.

### 2.2 Data Collection for PLE Cases

The study baseline timepoint (*T*_0_) was defined as the date of initial presentation when a diagnosis of PLE was made, whereas the post‐treatment follow‐up timepoint (*T*_1_) was defined as either the visit when the owner reported clinical improvement (in responders) or the visit when SDMA measurement was last performed (in non‐responders). Signalment data were recorded at *T*_0_, whereas clinical data recorded at both timepoints included clinical history, physical examination findings (including body weight, body condition score [BCS] and muscle condition score [MCS], when available), CBC, serum biochemistry (including SDMA), urinalysis (including UPCR), diagnostic imaging, and histopathology. During the study period, biochemical analyses were performed at an external laboratory (IDEXX laboratories, Wetherby, UK) including measurement of SDMA by liquid chromatography‐mass spectrometry (laboratory reference interval, 1–14 μg/dL), as previously described [5]. Treatments used in case management also were recorded, including dietary modification, immunosuppressive medications, other supportive medications (e.g., synbiotics, antiemetics, multimodal analgesia), and combinations of these. However, because of the retrospective nature of the study, treatment regimens were not standardized, such that exact agents used and timing of re‐evaluations were at the discretion of the attending clinicians.

### 2.3 Matched Control Group

To identify a group of matched control dogs (‘matched controls’) for each PLE case, the hospital database was searched for dogs that had been presented during the study period (from November 2017 to December 2020) and had CBC and serum biochemistry (including SDMA) performed. Dogs presented with urinary tract signs (e.g., hematuria, stranguria, dysuria, pollakiuria, polyuria), azotemia, or chronic gastrointestinal signs (e.g., vomiting or diarrhea > 3 weeks in duration) were excluded, as were dogs that had been treated with glucocorticoid or immunosuppressive medications within 3 months of presentation. Dogs with acute gastrointestinal signs (e.g., vomiting or diarrhea < 2 weeks in duration) within 2 weeks of presentation were not excluded. For each of the dogs in the PLE group, two dogs were selected chronologically in the order of presentation by matching age, breed, sex, and neuter status. Cases were matched as closely as possible by first allowing a deviation of 1 year in age, next allowing a difference in sex, and finally allowing a deviation of 2 years in age until the requisite control dogs were selected. From this control group, signalment, clinical history, physical examination findings (body weight, BCS, MCS), CBC, serum biochemistry, SDMA, urinalysis including UPCR, and diagnostic imaging findings were recorded.

To explore possible associations between SDMA and serum albumin and creatinine concentrations, a further group of dogs was recruited (‘SDMA‐albumin comparison group’). An a priori power analysis was conducted to determine the minimum sample size required for this control group, as described above. Using the Pearson's correlation coefficient obtained from the comparison between albumin and SDMA in the PLE‐Group (*R*_p_ = 0.470), an alpha of 0.05 and a power of 95%, a sample size of 52 would be required. However, to take into account unexpected variability within this group, we increased this sample size by approximately 50%, with a target group size of 70–80 dogs. From this control group, signalment, clinical history, physical examination findings (body weight, BCS, MCS), as well as SDMA, total protein (albumin and globulin), urea and creatinine concentrations were recorded.

### 2.4 Statistical Analysis

Initially, data were entered into an electronic spreadsheet (Excel for Mac, version 16.77.1, Microsoft) and checked for errors. Complete datasets were available from all PLE cases at *T*_0_, except for MCS, which was only available for 8/18 dogs. Some follow‐up data (*T*_1_) were available for 13/18 dogs, although some data points were missing: body weight and BCS were available for 12/13 dogs, MCS were available for 8/13, serum biochemistry data were available for 12/13, and SDMA was available for 10/13. For the matched control group, complete datasets were available from all dogs except for BCS, which was available for 29/36 dogs, and MCS, which was available for 7/36 dogs.

An online, open‐access statistical language and environment (R, version 4.3.1) [22] then was used for all statistical analyses with several additional packages including car (version 7.3.60 [23]), dplyr (version 7.3.60 [24]), effectsize (version 7.3.60 [25]), ggplot2 (version 7.3.60 [26]), ggsignif (version 7.3.60 [27]), jtools (version 7.3.60 [28]), lmtest (version 7.3.60 [29]), MASS (version 7.3.60 [30]), mctest (version 7.3.60 [31]), psych (version 7.3.60 [32]), pwr (version 7.3.60 [21]), and readexcel (version 7.3.60 [33]).

Initially, datasets of continuous data were assessed for normality using the Shapiro–Wilk test and by visually assessing Q–Q plots and histograms. Parametric statistical analyses were performed for datasets that were normally distributed. Those that were not normally distributed were either logarithmically or square‐root transformed and reassessed, with parametric tests being used if the dataset then was normally distributed. Where datasets were not normally distributed, either before or after transformation, non‐parametric statistical analyses were performed. Descriptive statistics for quantitative variables were expressed either as mean and SD if they were normally distributed or median (range) if they were not normally distributed. Categorical variables were expressed as number and percentage.

Simple statistical analyses were used to make comparisons between groups and between timepoints (*T*_0_ vs. *T*_1_) within the case group. Comparisons of categorical variables between groups were made using Fisher's exact test. Unpaired *t* tests were used for normally distributed continuous datasets (before or after transformation), whereas the Mann–Whitney test was used for datasets that were not normally distributed. Given that multiple comparisons were conducted, the false‐discovery rate was controlled by correcting *p* values using the Benjamini–Hochberg adjustment [34]. Effect size was calculated either using Hedge's G for comparisons involving *t* paired or unpaired *t* tests, or rank biserial for comparisons involving Mann–Whitney or Wilcoxon signed ranks tests. The Hedge's G effect size was interpreted according to previously described rules [35]: very small, < 0.01; small, 0.01–0.2; medium, 0.2–0.5; large, 0.5–0.8; very large, 0.8–1.2; 1.2–2.0, extremely large [36]. The rank biserial effect size was interpreted according to previously described rules [37]: < 0.05, extremely small; 0.05–0.10, very small; 0.10–0.20, small; 0.20–0.30, medium; 0.30–0.40, large; > 0.40 very large.

Simple and multiple linear regressions were used to determine variables associated with SDMA concentrations using the lm function in R. Explanatory variables tested included age, sex, neuter status, body weight, BCS, group (case or control), and serum concentrations of albumin, globulin, urea, and creatinine. The only between‐variable interactions tested were between sex and neuter status and between body weight and BCS; other interactions were deemed clinically irrelevant.

Initially, a series of simple models was created with SDMA as the outcome variable and single explanatory variables. A multiple regression model then was built that initially included all variables that were *p* < 0.2 on simple regression. This model then was refined in a backwards and forwards stepwise fashion, with the Bayesian information criterion (BIC) being used to select the model within the same family with the best generalizability (a measure of its goodness‐of‐fit compared with its complexity) [38, 39]. With this approach, the existing model was repeatedly refined with the addition or removal of variables until the model with the smallest BIC was identified. Model performance was assessed using adjusted *R* ^2^ and the associated *p* values, whereas model assumptions were tested in various ways. Normality of residuals was tested by visually inspecting histograms and Q–Q plots and using the Shapiro–Wilk test. Homogeneity of variance was tested using visual inspection of a plot of fitted values against the square root of the standardized residuals and with the Breusch–Pagan test. Cook's distance was used to identify possible influential datapoints; because none were identified, it was not necessary to consider removing any data points. Possible multicollinearity, in models containing multiple explanatory variables, was assessed using variance inflation factors (VIF). If multicollinearity was identified (e.g., VIF > 4), it was resolved by removing variables with the highest VIF. Separate multiple regression analyses also were performed, containing only one of the variables that had caused the multicollinearity.

A multiple regression model also was constructed to test associations between serum albumin and creatinine concentrations as explanatory variables and SDMA as the outcome variable using data from the SDMA‐albumin control population. The same methods, as described above, were used to test model assumptions and performance. To ensure that model assumptions were met and improved overall fit, this analysis required SDMA concentrations to be logarithmically transformed.

## 3 Results

### 3.1 Study Animals

**FIGURE 1:**
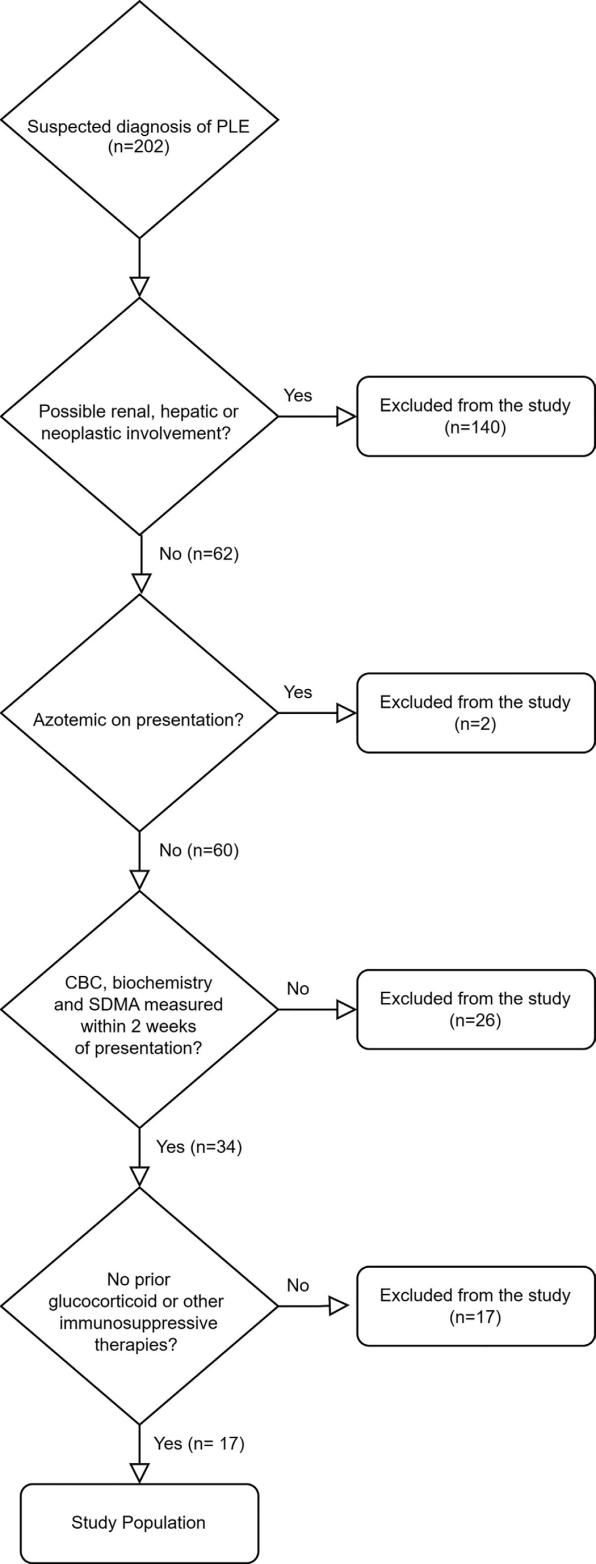
Flow diagram illustrating the selection criteria for the study. GI, gastrointestinal; PLE, protein‐losing enteropathy; SDMA, symmetric dimethylarginine.

Between November 2017 and December 2020, 202 dogs were diagnosed with PLE. Of these, 140 dogs were excluded because of insufficient evidence or incomplete clinical data to rule out hepatic, renal, or neoplastic conditions; two were excluded because of azotemia (serum creatinine concentration ≥ 1.4 mg/dL) at the time of SDMA measurement. Twenty‐six dogs were excluded because CBC, serum biochemistry, and SDMA had not been performed within 2 weeks of initial presentation, and 17 dogs were further excluded because they had received glucocorticoid or immunosuppressive medications within 3 months of presentation. As a result, 17 dogs met the eligibility criteria of the study (Figure 1). A database search determined that CBC, serum biochemistry, and SDMA data were available from 5574 dogs during the study period, and 34 matched controls were identified (Table 1).

**TABLE 1:** Signalment and clinical signs for the dogs in the study.

Variable	PLE cases	Matched controls	*p* ^a^	Effect size^b^
Number	17	34	—	—
Age (years)	7 (2 to 12)	7 (3 to 12)	0.83	0.06 (very small)
Sex			0.33	—
Male intact	1 (6%)	3 (9%)		
Male neutered	5 (29%)	9 (26%)		
Female intact	1 (6%)	3 (9%)		
Female neutered	10 (%)	19 (56%)		
Breed			—	—
Border collie	2 (12%)	4 (12%)		
Mixed breed	4 (22%)	9 (26%)		
Pug	2 (12%)	4 12%)		
Staffordshire bull terrier	2 (12%)	4 (12%)		
Bernese mountain dog	1 (6%)	2 (6%)		
Bichon frisé	1 (6%)	2 (6%)		
Boston terrier	1 (6%)	2 (6%)		
Dachshund	1 (6%)	2 (6%)		
Labrador retriever	1 (6%)	2 (6%)		
Newfoundland	1 (6%)	1 (3%)		
Rottweiler	1 (6%)	2 (6%)		
Bodyweight (kg)	19.3 (3.4 to 56.0)	23.6 (5.4 to 77.3)	0.65	0.12 (small)
Body condition score (/9)	4 (2 to 7)	5 (3 to 8)	**0.001**	0.66 (very large)

*Note:* Bold value indicate statistically significant.

Abbreviation: PLE, protein‐losing enteropathy.

^a^ Age data were compared by group with a two‐sample *t* test, after square‐root transformation; difference in sex and neuter status was assessed with Fisher's exact test; body weight and body condition score were assessed with Mann–Whitney tests; the false‐discovery rate was controlled by correcting all *p* values using the Benjamini–Hochberg adjustment [40].

^b^ Effect size used for age and weight was Hedge's G, and interpreted according to previously described rules [41]: very small, < 0.01; small, 0.01–0.2; medium, 0.2–0.5; large, 0.5–0.8; very large, 0.8–1.2, 1.2–2.0, extremely large; effect size used for body weight and body condition score was the rank biserial, interpreted according to a previous study [42] < 0.05, extremely small; 0.05–0.10, very small; 0.10–0.20, small; 0.20–0.30, medium; 0.30–0.40, large; > 0.40 very large.

### 3.2 Comparisons Between PLE Cases and Matched for Baseline Variables

No differences in age, sex, neuter status, and body weight were identified between the PLE cases and controls (Table 1; *p* > 0.3 for all, small or very small effects). However, BCS was lower in PLE cases compared with matched controls (Table 1; *p* = 0.001, very large effect). Muscle condition score only was recorded in 8 cases (A: 1; B: 4; C: 2; D: 1) and seven controls (A: 2: B: 5); given the small numbers, statistical analysis was not conducted.

Of the 17 PLE cases, clinical signs included: weight loss (7/17; 41%), hyporexia (5/17; 29%), cavitary effusion (6/17; 35%), vomiting (4/17; 24%), and lethargy (3/17; 18%). Further information is available in the Supporting Information S1.

### 3.3 Diagnostic Investigations, Treatment, and Follow‐Up in Dogs of the PLE Case Group

At the initial visit (*T*_0_), 10 (59%) and 11 (65%) of the dogs in the PLE case group had urinalysis and UPCR performed, respectively, with urinalysis and UPCR results being available from the referring veterinarian in an additional 5 (50%) and 6 (35%) dogs, respectively. Serum trypsin‐like Immunoreactivity (TLI) was measured in 4 (24%) dogs, whereas serum cobalamin and folate concentrations were measured in 10 (59%) and 9 (53%) dogs, respectively. Basal cortisol concentration was measured in 7 (41%) of the dogs in the case group. Radiography, abdominal ultrasonography, and computed tomography were performed in 10 (59%), 15 (88%) and 3 (18%) dogs, respectively. Endoscopic biopsy samples of the gastrointestinal tract and histopathological examination were performed in 11 cases (65%). Details of clinical findings are summarized in the Supporting Information S1.

Four dogs (24%) received dietary modification alone, five dogs (29%) received glucocorticoid treatment and chlorambucil, and seven dogs (41%) received dietary modification, glucocorticoids, and chlorambucil. No other immunosuppressive drugs were used. Three dogs (18%) received concurrent antimicrobial treatment (metronidazole in three dogs and oxytetracycline in one dog).

Twelve of the 17 PLE cases were evaluated at the referral center for follow‐up (*T*_1_). The median duration from initial presentation (*T*_0_) to post‐treatment follow‐up (*T*_1_) was 34 days (range, 9 to 407 days). Investigations performed in these cases included CBC (9 dogs) and serum biochemistry (12 dogs), with SDMA concentration being available in nine dogs. Details of findings at follow‐up are provided in the Supporting Information S1.

#### 3.3.1 Clinical Response in Cases With PLE

The owners of 10/12 (83%) dogs reported clinical improvement at *T*_1_, of which 3/12 (25%) were reported to have complete resolution of clinical signs. Body weight (*T*_0_, 19.3 ± 13.66 kg; *T*_1_ 21.2 ± 15.04 kg; *p* = 0.65; rank biserial 0.24 [medium effect]) and BCS (*T*_0_ 4 [2–7]; *T*_1_ 4 [2–6]; *p* = 0.17, rank biserial 0.42 [large effect]) did not differ between initial presentation and follow‐up.

#### 3.3.2 Comparisons of SDMA and Key Biochemical Analytes Between Cases and Controls

**FIGURE 2:**
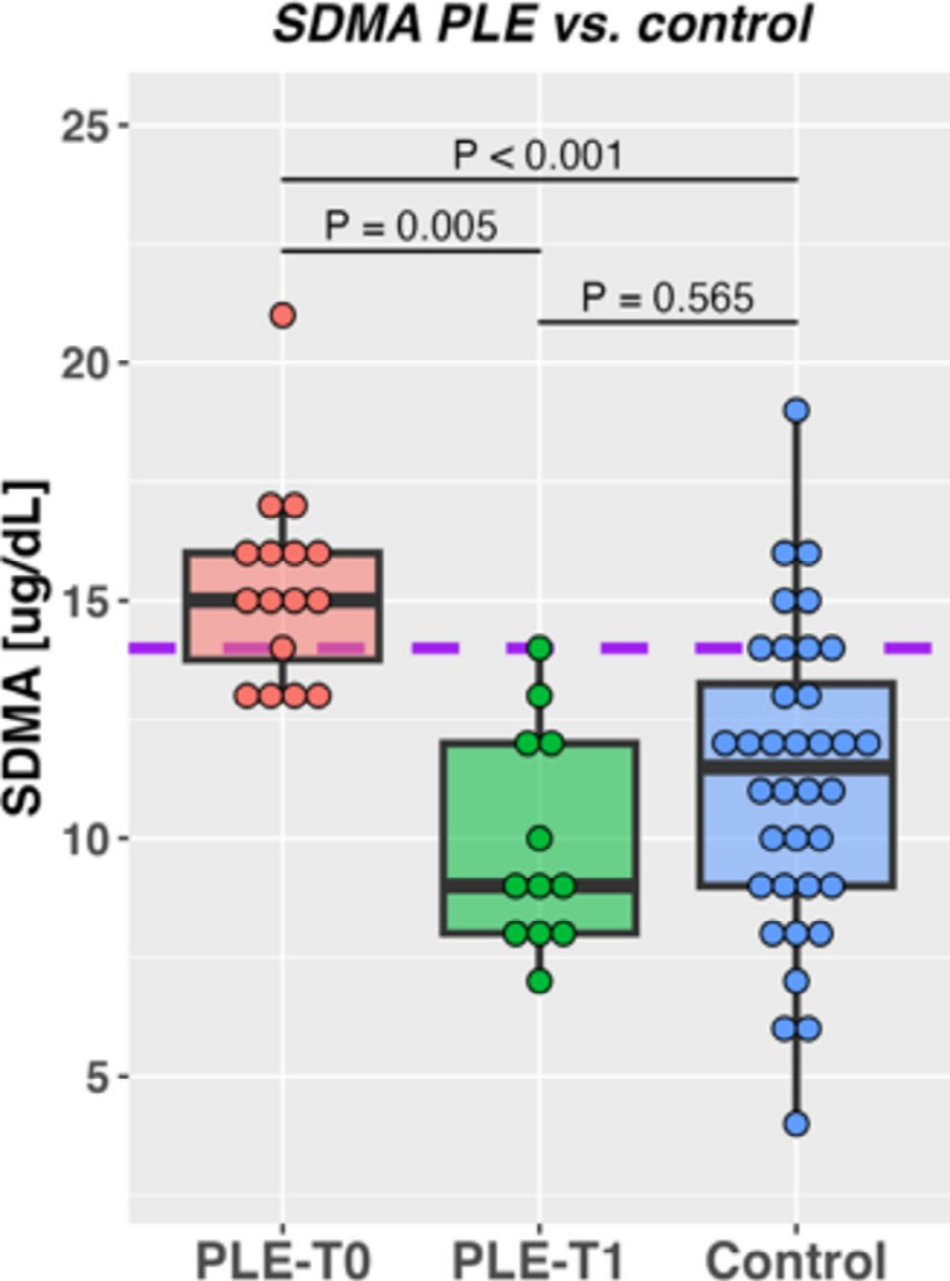
Comparison of symmetric dimethylarginine (SDMA) concentrations in 17 protein‐losing enteropathy (PLE) cases at original presentation (PLE‐*T*_0_) and following treatment (PLE‐*T*_1_, *n* = 9), and 34 matched controls (Control). The purple dotted line represents the upper limit of the reference range for the SDMA assay.

Results of SDMA and other biochemical analytes are shown in Table 2. At initial presentation, SDMA concentration was higher in PLE cases compared with matched controls (PLE case *T*_0_ vs. control: *p* < 0.001, Hedge's G, 1.48 [very large effect]; Figure 2) and was higher than the upper limit of the reference interval in 11/17 dogs (65%). At follow up, *T*_1_, SDMA concentration had decreased (PLE case *T*_0_ vs. T_1_: *p =* 0.01, Hedge's G, 1.31 [very large effect]), remained above the reference range in only 1/9 (11%), and was no longer different from the matched controls (PLE case *T*_1_ vs. control: *p = 0.57*; Hedge's G, 0.21 [small effect]).

**TABLE 2:** Comparison of SDMA and key biochemical analytes in protein‐losing enteropathy (PLE) cases and matched controls.

Variable	PLE cases		Matched controls	Statistical analyses		
	*T*_0_	*T*_1_		Comparison	*p* ^a^	Effect size^b^
SDMA (μg/dL)	15.2 (2.01)	10.3 (2.78)	11.0 (3.13)	Case *T*_0_ vs. control	< **0.001**	1.48 (very large)
				Case *T*_1_ vs. control	0.57	0.21 (small)
				Case *T*_0_ vs. case *T*_1_	**0.01**	1.31 (very large)
Urea (mg/dL)^c^	15.5 (4.31)	19.5 (8.92)	14.5 (5.96)	Case *T*_0_ vs. control	0.41	0.27 (small)
				Case *T*_1_ vs. control	0.07	0.27 (small)
				Case *T*_0_ vs. case *T*_1_	0.32	0.32 (small)
Creatinine (mg/dL)	0.81 (0.24)	0.58 (0.331)	0.85 (0.256)	Case *T*_0_ vs. control	0.57	0.18 (very small)
				Case *T*_1_ vs. control	**0.04**	0.96 (large)
				Case *T*_0_ vs. case *T*_1_	0.07	0.62 (medium)
Albumin (mg/dL)	1.60 (0.512)	2.29 (0.649)	2.96 (0.494)	Case *T*_0_ vs. control	< **0.001**	2.68 (huge)
				Case *T*_1_ vs. control	**0.002**	1.23 (very large)
				Case *T*_0_ vs. case *T*_1_	**0.003**	1.14 (large)
Globulins (mg/dL)	2.09 (0.380)	2.33 (0.496)	3.46 (0.530)	Case *T*_0_ vs. control	< **0.001**	2.79 (huge)
				Case *T*_1_ vs. control	< **0.001**	2.13 (huge)
				Case *T*_0_ vs. case *T*_1_	0.08	0.57 (medium)

*Note:* SDMA and biochemical analytes are reported as mean (standard deviation). Bold values indicate statistically significant.

Abbreviation: SDMA, Serum symmetric dimethylarginine concentration.

^a^ Comparisons made with 2‐sample *t* test; the false‐discovery rate was controlled by correcting all *p* values using the Benjamini‐Hochberg adjustment [40].

^b^ Effect size used for age and bodyweight was Hedge's G, and interpreted according to the previously described rules [41]: very small, 0.01; small, 0.2; medium, 0.5; large, 0.8; very large, 1.2; 2.0, extremely large.

^c^ Urea concentrations were logarithmically‐transformed before analysis in order to ensure that the data were normally‐distributed.

At initial presentation, serum creatinine concentrations in the PLE cases did not differ from concentrations in the matched controls (PLE case *T*_0_ vs. control: *p* = 0.57; Hedge's G, 0.18 [small effect]; Table 2) and none were above the upper limit of the reference interval. Serum creatinine concentrations in the case group had not changed significantly by the follow‐up visit (PLE cases at *T*_0_ vs. T_1_: *p =* 0.07; Hedge's G, 0.62 [small effect]), but differed from serum creatinine concentrations in matched control dogs at this stage (PLE case *T*_1_ vs. control: *p* = 0.04; Hedge's G, 0.96 [large effect]). No significant differences were found in urea concentrations (*p* > 0.072; Table 2).

When measured at initial presentation, concentrations of both albumin (case *T*_0_ vs. control: *p* < 0.001, Hedge's G, 2.68 [extremely large effect]) and globulins (PLE case *T*_0_ vs. control: *p* < 0.001; Hedge's G, 2.79 [extremely large effect]) were less than their respective concentrations in control dogs. By the follow‐up visit, serum albumin concentration in the case group had increased (PLE case *T*_0_ vs. *T*_1_: *p* = 0.003; Hedge's G, 1.14 [large effect]) but remained less than in the control dogs (PLE case *T*_1_ vs. control: *p* = 0.002; Hedge's G, 1.23 [very large effect]). In contrast, no change was identified in serum globulin concentration in cases between initial presentation and follow‐up (PLE case *T*_0_ vs. *T*_1_: *p* = 0.08; Hedge's G, 0.57 [medium effect]), and follow‐up concentrations remained less than concentrations in controls (PLE case *T*_1_ vs. control: *p* < 0.001; Hedge's G, 2.13 [extremely large effect]).

#### 3.3.3 Simple and Multiple Linear Regression Modeling to Determine Variables Associated With SDMA

Details of simple linear regression analyses are given in Table 3. Of the variables assessed, group (PLE case vs. control; *p* < 0.001), serum albumin concentration (*p* < 0.001), serum globulin concentration (*p* < 0.001) and serum creatinine concentration (*p* = 0.091) met the threshold (*p* < 0.2) for inclusion in the initial multiple regression model. However, multicollinearity was evident in models that contained both group and serum albumin concentration. As a result, refinement by backwards and forwards stepwise regression was conducted twice, by including either group (multiple regression model 1) or serum albumin concentration (multiple regression model 2) with the other qualifying variables. For multiple regression model 1, the best fit (adjusted *R* ^2^, 0.404; *p* < 0.001) and generalizability (BIC, 257) were obtained when two predictor variables were included: group and creatinine, with SDMA concentrations being positively associated with creatinine and PLE (Table 4). For multiple regression model 2, the best fit (adjusted *R* ^2^, 0.429; *p* < 0.001) and generalizability (BIC, 255) were obtained when two predictor variables were included, creatinine and albumin, with SDMA concentration being positively and negatively associated with serum creatinine and albumin concentrations, respectively (Table 5).

**TABLE 3:** Simple linear regression analysis to determine variables associated with SDMA concentrations in protein‐losing enteropathy (PLE) and matched controls.

Parameter	Estimate^a^	95%‐CI^a^	Adjusted^b^ *R* ^2^	BIC^c^	*p*
Age (years)^e^	0.052	−0.336, 0.441	0.019	281	0.79
Sex					
Female	Ref	—	—	—	—
Male	0.394	−1.650, 2.438	0.017	281	0.70
Neuter status					
Intact	Ref	—	—	—	—
Neutered	0.340	−2.348, 3.029	0.019	2810.800	
Bodyweight (per kg)	0.015	−0.044, 0.074	0.015	281	0.61
Body condition score (per unit)^d^	−0.857	−1.538, −0.176	0.110	244	**0.02**
Group^e^					
Matched controls	Ref	—	—	—	—
PLE cases	4.124	2.553, 5.917	0.330	260	< **0.001**
Albumin (per mg/dL)	−2.517	−3.489, −1.544	0.342	260	< **0.001**
Globulins (per mg/dL)	−1.970	−3.047, −0.894	0.200	269	< **0.001**
Urea (per mg/dL)	0.109	−0.070, 0.288	0.010	280	0.23
Creatinine (per μg/dL)	3.293	−0.541, 7.127	0.038	279	0.09

*Note:* Bold values indicate statistically significant.

Abbreviation: SDMA, Serum symmetric dimethylarginine concentration.

^a^ Estimate and 95% confidence interval of the regression coefficient for the predictor variable; the coefficient represents the expected change in SDMA for each unit increase in the predictor.

^b^ Model performance was assessed by calculating *R* ^2^ adjusted for the number of predictors in the model.

^c^ Model generalizability is determined using the Bayesian information criterion (BIC), with models having the best fit having lower BIC [43, 44]. The BIC values can only be compared within the same family of models.

^d^ Body condition scoring was performed using the nine‐unit system [38, 39]. Although the smallest BIC was returned, this result was because eight dogs were dropped due to missing data.

^e^ Cases were the 17 dogs with protein‐losing enteropathy, whereas controls were the 34 dogs that were age, sex, neuter, and breed‐matched to the cases. *p*‐values in bold are those that met the threshold for inclusion in the multiple linear regression modeling (Table 4).

**TABLE 4:** Best‐fit multiple linear regression model 1 (containing group and other variables) assessing variables associated with SDMA concentrations in protein‐losing enteropathy (PLE) cases and matched controls.

Parameter	Estimate^a^	95%‐CI^a^	Adjusted^b^ *R* ^2^	BIC^c^	*p*
Final model details	—	—	0.404	257	**0.01**
Predictor variables					
Group^d^					
Matched controls	Ref	—	—	—	—
PLE cases	4.420	2.827, 6.013	—	—	< **0.001**
Creatinine (per μg/dL)	4.026	0.997, 7.056	—	—	**0.01**

*Note:* Bold values indicate statistically significant.

Abbreviation: SDMA, Serum symmetric dimethylarginine concentration.

^a^ Estimate and 95% confidence interval of the regression coefficient for the predictor variable; the coefficient represents the expected change in SDMA for each unit increase in the predictor.

^b^ Model performance was assessed by calculating *R* ^2^ adjusted for the number of predictors in the model.

^c^ Model generalizability is determined using the Bayesian information criterion (BIC), with models having the best fit having lower BIC [43, 44]. The BIC values can only be compared within the same family of models.

^d^ Cases were the 17 dogs with protein‐losing enteropathy, whereas controls were the 34 dogs that were age, sex, neuter, and breed‐matched to the cases [38, 39]. SDMA: Serum symmetric dimethylarginine concentration.

**TABLE 5:** Best‐fit multiple linear regression model 2 (containing albumin and other variables) assessing variables associated with SDMA concentrations in protein‐losing enteropathy (PLE) cases and matched controls.

Parameter	Estimate^a^	95%‐CI^a^	Adjusted^b^ *R* ^2^	BIC^c^	*p*
Final model details	—	—	0.429	255	< **0.001**
Predictor variables					
Albumin (per mg/dL)	−2.667	−3.580, −1.754	—	—	< **0.001**
Creatinine (per μg/dL)	4.286	1.310, 7.262	—	—	**0.01**

*Note:* Bold values indicate statistically significant.

Abbreviation: SDMA, Serum symmetric dimethylarginine concentration.

^a^ Estimate and 95% confidence interval of the regression coefficient for the predictor variable; the coefficient represents the expected change in SDMA for each unit increase in the predictor.

^b^ Model performance was assessed by calculating *R* ^2^ adjusted for the number of predictors in the model.

^c^ Model generalizability is determined using Bayesian information criterion (BIC), with models having the best fit having lower BIC [43, 44]. The BIC values can only be compared within the same family of models. SDMA: Serum symmetric dimethylarginine concentration.

#### 3.3.4 Further Assessment of Association Between SDMA and Albumin Concentrations in a SDMA –Albumin Comparison Group

In multiple regression modeling, the fits of best‐fit model 1 (which contained group and creatinine) and best‐fit model 2 (which contained albumin and creatinine) were similar (i.e., BIC within 2 units). It was suspected that the albumin association was the result of the group effect, considering the marked hypoalbuminemia that PLE can cause, although this suspicion could not be confirmed with the available data. Therefore, a separate group of 76 dogs was randomly selected to evaluate the association between albumin and SDMA (albumin‐SDMA group; Table 6). A multiple linear regression model was created with the same variables as for best‐fit model 2 (Table 5). To ensure model assumptions (normal distribution of residuals) were met, SDMA concentrations were first logarithmically transformed (Table 7), which also improved model fit (SDMA as predictor variable: adjusted *R* ^2^, 0.095; BIC, 461; log[SDMA] as predictor variable: adjusted *R* ^2^, 0.117; BIC, 55). In this model, a significant effect of creatinine (*p* = 0.002) but not albumin (*p* = 0.15) was observed.

**TABLE 6:** Details of the SDMA –albumin comparison group used for examining associations between SDMA and both albumin and creatinine concentrations.

Variable	Cases
Number	76
Age (years)	7 (0 to 14)
Sex	
Male intact	10 (13%)
Male neutered	26 (34%)
Female intact	8 (11%)
Female neutered	32 (42%)
Breed	American bulldog, Beagle 2, Bedlington terrier, Border terrier 2, Border collie, Boston terrier, Boxer 5, Bulldog, Bullmastiff, Cairn terrier, CKCS 4, Chihuahua 2, Cocker spaniel 5, Dachshund, Dalmatian, Doberman, English springer spaniel 3, Fox terrier, Golden retriever 2, Greyhound, Hungarian vizla 2, Irish setter 2, Labrador retriever 5, Lhasa apso, Miniature dachshund, Miniature schnauzer, Mixed breed 13, Pug 2, Rottweiler 2, Presa Canario, Siberian husky, Spanish terrier, Staffordshire bull terrier 3, Yorkshire terrier, West Highland white terrier, Whippet
Diagnoses	AGASACA 4, AKI grade IV, alopecia, ARVC, Aspergillosis, atopic dermatitis, IVDD, carcinoma, cerebellitis 2, ceruminous cell carcinoma, cholecystitis, cruciate ligament disease 4, cutaneous lymphoma, diabetes Insipidus, dilated cardiomyopathy, epilepsy 2, exertional rhabdomyolysis, gall bladder mucocele, heart‐based mass, hepatic dysfunction, histiocytic sarcoma, hyperadrenocorticism, hypoadrenocorticism, intracranial lesions 2, intracranial mass, intervertebral disc disease, Leishmaniosis and Ehrlichiosis, discospondylitis, lymphoma 5, mast call tumor 4, mediastinal tumor, meningitis of unknown origin, myxomatous mitral valve disease, osteoarthritis, osteosarcoma, obesity, oral fibrosarcoma, osteosarcoma, paraphimosis, patent ductus arteriosis, phenobarbital‐responsive sialoadenitis, pneumonia, portosystemic shunt, prostatic carcinoma, PSOM, salivary gland tumor, seizures, severe multifocal pyogranulomatous panniculitis, soft‐tissue sarcoma 3, SRMA, transitional cell carcinoma, thyroid carcinoma, ureteral bypass
Bodyweight (kg)	18.7 (2.2 to 52.5)
Body condition score (/9)	5 (2 to 9)
SDMA (μg/dL)	13.3 (4.80)
Creatinine (μg/dL)	0.80 (254)
Albumin (mg/dL)	3.05 (0.440)

*Note:* Continuous variables are reported as either mean (standard deviation) or median (range), whilst categorical variables are reported as number (percentage).

Abbreviations: AGASACA, Apocrine gland anal sac adenocarcinomas; ARVC, Arrhythmogenic right ventricular cardiomyopathy; PSOM, Primary Secretory Otitis Media; SRMA, Steroid responsive meningitis‐arteritis.

## 4 Discussion

In dogs, breed, body fat percentage, and lymphoma are extrarenal factors associated with increased SDMA concentration [12, 45, 46]. In humans, multiple conditions including IBD are associated with increased serum SDMA concentrations because of extrarenal factors such as endothelial dysfunction and changes in protein metabolism [13, 14, 15, 17, 18]. Based on these observations, we hypothesized that dogs diagnosed with PLE would also have increased serum SDMA concentrations. Our primary objective was to evaluate SDMA concentration in nonazotemic dogs diagnosed with PLE. Nearly two‐thirds of dogs in the PLE case group had SDMA concentrations higher than the reference interval without concurrent azotemia.

The correlation between SDMA and creatinine as surrogate markers for GFR has been well documented [3, 4, 5, 6, 7]. However, recent studies have identified discordance between these markers in some dogs [3, 6, 7, 40, 41]. In cases where SDMA is increased despite normal serum creatinine concentrations, it remains unclear whether this observation reflects SDMA's higher sensitivity in detecting early decreases in GFR or its poor specificity as a renal biomarker. Conversely, a previous longitudinal study reported an approximately 12% likelihood of dogs with normal SDMA developing increased serum creatinine concentrations over 2 years [42, 47]. Additionally, 48 of the 8088 dogs exhibited increased serum creatinine concentration with normal SDMA concentration after a prior increase in SDMA [42]. The authors attributed these findings to day‐to‐day GFR variability [43], but it is also possible that extrarenal factors may have influenced SDMA. For example, overexpression of protein arginine methyltransferase 5 (PRMT5) in lymphoma has been proposed as a potential contributor to increased SDMA in dogs [44, 48]. As a type II protein arginine methyltransferase, PRMT5 is a well‐known epigenetic enzyme responsible for catalyzing SDMA formation [49]. In turn, SDMA competitively inhibits intracellular uptake of L‐arginine, an essential substrate for the synthesis of nitric oxide (NO), via the inhibition of cationic amino acid transporter [50, 51]. In inflammatory bowel disease (IBD) of humans, decreased NO contributes to microcirculatory dysfunction, mucosal ulceration, and impaired wound healing [52, 53, 54, 55]. Upregulation of PRMT5 and a subsequent increase in SDMA concentration have been documented in people with IBD and in experimental murine models of IBD [56, 57, 58, 59]. Furthermore, in vivo studies in humans have documented increased endothelial dysfunction markers, including SDMA, in IBD patients [17, 60]. Although IBD in humans and PLE in dogs are not directly comparable, similar mechanisms may influence SDMA concentrations in dogs.

Interestingly, PRMT5 also regulates lipid metabolism in people [61, 62], and negative associations between PRMT5 and dogs and people with obesity have been observed [46, 63]. Given albumin's role in lipoprotein metabolism [64, 65], hypoalbuminemia secondary to increased enteric loss in dogs with PLE might alter the rate of hepatic protein (particularly albumin) and lipid synthesis, as well as albumin catabolism and urinary clearance [66]. The precise physiological interaction between albumin and SDMA is yet to be determined, considering that albumin and SDMA concentrations were not associated in the second control group. However, PRMT5 and SDMA are thought to regulate protein‐to‐protein interaction through the Tudor domains [67, 68]. The association between SDMA and both protein turnover and lipid metabolism requires further research, which might incidentally explain why SDMA concentration decreases after nutritional intervention with functional lipids and antioxidants in nonazotemic dogs [69].

Limited research exists on renal function in dogs with PLE. One study reported serum creatinine concentrations within or below the reference range [70], whereas another found higher serum creatinine concentrations in dogs with food‐responsive PLE compared with those with immunosuppressant‐responsive or non‐responsive PLE, although renal function was not directly assessed [71]. In our study, serum creatinine concentration was used as a surrogate marker for GFR, but it is arguably less sensitive to changes in GFR than SDMA [3]. Increases in SDMA might have resulted from changes in GFR below the detection limit of serum creatinine concentration. Considering recent information on the kidney‐gut axis [72], future studies employing direct GFR measurements using iohexol or inulin would be required to clarify whether SDMA concentrations in PLE dogs are affected by decreased GFR.

We aimed to identify clinicopathological variables associated with SDMA concentration in PLE dogs and matched controls. Multiple regression analysis identified two best‐fit models, one including creatinine and group status and another including creatinine and albumin. Given that hypoalbuminemia (albumin 2.63 g/dL [26.3 g/L]) was required for the diagnosis of PLE, significant multicollinearity was found between the group variable and albumin concentration. Therefore, a control group was recruited to assess SDMA‐albumin associations independent of PLE. No association was found between albumin and SDMA concentration in this group, confirming that the initial SDMA‐albumin correlation was driven by systematic differences in albumin between groups [38, 39].

**TABLE 7:** Multiple linear regression model assessing associations between log(serum SDMA)^a^ and both albumin and creatinine, in a the SDMA‐albumin comparison group.

Parameter	Estimate^b^	95%‐CI^b^	Adjusted^c^ *R* ^2^	BIC^d^	*p*
Final model details	—	—	0.117	55	**0.01**
Predictor variables					
Albumin (per mg/dL)	−0.120	−0.287, 0.046	—	—	0.15
Creatinine (per μg/dL)	0.474	0.186, 0.762	—	—	**0.002**

*Note:* Bold values indicate statistically significant.

^a^ Serum symmetric dimethylarginine concentration was logarithmically transformed because this ensured that model assumptions were met and improved model fit.

^b^ Estimate, and 95% confidence interval, of the regression coefficient for the predictor variable (SDMA); given that the outcome variable was logarithmically‐transformed, the coefficients represent the expected change in log(serum SDMA) per unit change in the predictor variable; taking creatinine as an example, each 1.0 μg/dL increase is associated with an estimated increase in SDMA of exp.(0.474) = 1.61 mg/dL.

^c^ Model performance is assessed by calculating *R* ^2^ adjusted for the number of predictors in the model.

^d^ Model generalizability is determined using Bayesian information criterion (BIC), with models having the best fit having lower BIC [43, 44]. The BIC values can only be compared within the same family of models.

In our analysis, serum creatinine concentrations correlated better with SDMA concentrations after logarithmic transformation, a finding that contrasts with a previously documented linear association between SDMA and creatinine in dogs with X‐linked inherited nephropathy [5]. One possible explanation for this discrepancy may be the exclusion of azotemic dogs in our study, limiting the range for comparison between the two analytes. In fact, no associations between SDMA and serum creatinine concentration were seen in the unaffected littermates in the previous study [5].

We also assessed SDMA changes after treatment in nine dogs with PLE. Because of the retrospective study design, timings of follow‐ups and treatments used were not standardized, and follow‐up SDMA measurements were not available for all dogs. Despite these limitations, SDMA concentration decreased significantly post‐treatment. Because the mechanism of the increase in SDMA in dogs with PLE is not known, it is also not clear why SDMA concentrations decreased after treatment. If an extra‐renal mechanism such as upregulation of PRMT5 were responsible, resolution of such an abnormality might explain the observed decrease, but additional studies are needed to confirm this hypothesis.

In addition to decreases in SDMA concentration, albumin concentrations increased after treatment, whereas globulin concentrations remained unchanged. This pattern has been reported anecdotally but contradicts previous literature [2, 19]. Decreased globulin concentration associated with glucocorticoid treatment has been reported in human medicine, but not in the veterinary literature [73, 74].

Another interesting finding was the decrease in serum creatinine concentration after treatment. Despite its frequent use in veterinary medicine to assess renal function, creatinine is influenced by extra‐renal factors such as lean body mass, age, breed, and feeding status [9, 75, 76, 77, 78, 79]. The observed decrease in serum creatinine concentration may reflect muscle mass loss caused by glucocorticoid therapy [80], given that almost all (12/13, 92%) of the dogs returning for follow‐up had received a glucocorticoids. Muscle condition score could have provided supportive evidence, but assessments were infrequent, preventing statistical analysis. Alternatively, decreases in serum creatinine concentration may indicate improved GFR after successful treatment, although this explanation seems less likely given that serum creatinine concentrations in PLE dogs typically remained within the reference range in previous studies [70].

Our study had several limitations. Firstly, the retrospective nature of the study meant that the diagnosis of PLE and subsequent treatment protocols were not standardized. As a result, diagnosis was decided by the attending clinician, and it included some cases where gastrointestinal biopsies had not been performed. Other possible causes of hypoalbuminemia (e.g., endocrine, hematological, renal and hepatic) were eliminated at the discretion of the attending clinician. Biopsies could have enabled possible associations between SDMA concentration and certain histological patterns to be identified. Previous studies, however, have identified variability in interpretation among pathologists, poor correlation among histological findings and types of disease, and an inability to predict clinical outcome [81, 82, 83].

A second limitation was the fact that response to treatment was not objectively assessed, and in some cases, no response was documented. Furthermore, in cases that did respond, the timing of the follow‐up visit (*T*_1_) was defined by the visit where the owner reported improvement in clinical signs. Such responses are likely subjective and might be affected by a possible placebo effect influencing owner perception. Finally, no validated disease severity scoring system was used, although limiting cases to those with PLE allowed albumin improvements to serve as an objective treatment response marker. Additional studies assessing the correlation between SDMA concentration and validated scoring systems in dogs with chronic enteropathy are recommended.

A third limitation was that because of strict eligibility criteria, we did not have sufficient cases to meet the intended sample size, based on our a priori power analysis (e.g., 17 vs. 20 cases). Whereas the study was technically underpowered, many significant findings were identified, with large effect sizes. Additionally, our a priori power analysis used an expected power of 0.95 at *α* = 0.05, more stringent than commonly applied thresholds (e.g., 0.90 or 0.80), making Type II errors less likely. Similarly, as discussed, serum creatinine concentration was used as a surrogate marker for GFR.

Finally, the short follow‐up period precluded assessment of long‐term SDMA changes and treatment response. It would be useful to determine whether the decreases in SDMA associated with treatment were maintained and whether SDMA could be used as a biomarker to identify relapse.

In conclusion, nonazotemic dogs presented with PLE had significantly increased SDMA concentrations compared with matched controls, and concentrations decreased after treatment. Further research is needed to elucidate the mechanisms underlying these changes and to determine whether extrarenal factors also influence SDMA.

## Disclosure

Authors declare no off‐label use of antimicrobials.

## Ethics Statement

Approved by the Veterinary Research Ethics Committee at the University of Liverpool. approval number: VREC1161a. Authors declare human ethics approval was not needed.

## Conflicts of Interest

Alexander J. German is an employee of the University of Liverpool, but his position is financially supported by Royal Canin. He has also received financial remuneration and gifts for providing educational material, speaking at conferences and consultancy work. All such remuneration has been for projects unrelated to the current work. The other authors declare no conflicts of interest.

## Supporting information


**Data S1.** Supporting Information.
